# Supplementary material for: Breast cancer histopathology image-based gene expression prediction using spatial transcriptomics data and deep learning
Source: Sci Rep. 2023 Aug 21;13:13604. doi: 10.1038/s41598-023-40219-0 (PMC10442349; doi:10.1038/s41598-023-40219-0)
Supplement: Supplementary file 1 — Supplementary Information. [file 41598_2023_40219_MOESM1_ESM.pdf]

Breast cancer histopathology image-based gene expression prediction using spatial transcriptomics data and deep learning

Md Mamunur Rahaman[1], **Ewan K. A. Millar**[2, 3, 4], **and Erik Meijering**[1,*]

[1]School of Computer Science and Engineering, University of New South Wales, Kensington, NSW 2052, Australia
[2]Department of Anatomical Pathology, NSW Health Pathology, St. George Hospital, NSW 2217, Australia
[3]St. George and Sutherland Clinical School, University of New South Wales, Kensington, NSW 2052, Australia
[4]Faculty of Medicine & Health Sciences, Sydney Western University, Campbelltown, NSW 2560, Australia
[*]erik.meijering@unsw.edu.au

SUPPLEMENTARY INFORMATION

Table S1. Top 10 predicted genes by our framework using different models without AuxNet. The numbers are median PCC values on the heldout test patient ID BC23903. Here, to save space, "EfficientNet" is abbreviated to "ENet", and "Inception-v3" to "Incep-v3". Bold indicates best performance (highest PCC) per gene and underlined indicates second-best.

Genes	ResNet101	Incep-v3	ENet-b0	ENet-b1	ENet-b2	ENet-b3	ENet-b4	ENet-b5	ViT-B16	ViT-B32
B2M	0.5715	0.5685	0.5746	0.5695	0.5616	0.5497	0.5798	**0.5922**	<u>0.5887</u>	0.4698
ACTG1	0.5088	0.5480	0.6087	0.5703	<u>0.6090</u>	0.5305	0.5909	**0.6142**	0.5468	0.4414
ACTB	0.5396	0.5385	0.5171	0.5293	0.5811	**0.5865**	0.4980	<u>0.5859</u>	0.5290	0.4470
TMSB10	0.4410	0.4018	0.3313	0.3662	0.5438	<u>0.5679</u>	0.4717	**0.6063**	0.1378	0.3972
GNAS	0.5237	0.5613	**0.5952**	0.5581	0.5541	0.5089	0.5654	<u>0.5819</u>	0.5093	0.5038
PTMA	0.5402	0.5099	0.5155	0.5332	**0.5530**	0.5229	0.5305	<u>0.5494</u>	0.4796	0.4047
PTPRF	0.4503	0.4852	<u>0.5327</u>	0.4970	0.5171	0.5111	0.4957	**0.5531**	0.4404	0.3742
ERBB2	0.4635	0.3730	0.4977	0.4494	0.5406	<u>0.5414</u>	0.5391	**0.5502**	0.4056	0.3994
PRDX1	0.4364	0.4544	0.4392	0.3734	<u>0.5009</u>	0.4826	0.4954	**0.5091**	0.4538	0.4245
TMSB4X	0.4544	0.4139	0.4491	0.4259	0.5491	0.5072	<u>0.5523</u>	**0.5674**	0.3665	0.4450

Table S2. Results of the cross-validation (mean +/- stdev over the 5 folds) with AuxNet.

Model	Validation data	
	aMAE	aRMSE
ResNet101 + AuxNet	0.9001 ± 0.0417	1.1473 ± 0.0621
Inception-v3 + AuxNet	0.8629 ± 0.0713	1.0612 ± 0.0905
EfficientNet-b0 + AuxNet	0.8274 ± 0.0469	1.0405 ± 0.0762
EfficientNet-b1 + AuxNet	0.8287 ± 0.0409	1.0375 ± 0.0554
EfficientNet-b2 + AuxNet	0.8452 ± 0.0667	1.0516 ± 0.0493
EfficientNet-b3 + AuxNet	0.8674 ± 0.0617	1.1021 ± 0.1575
EfficientNet-b4 + AuxNet	0.9334 ± 0.1554	1.3574 ± 0.4783
EfficientNet-b5 + AuxNet	0.8588 ± 0.0823	1.1314 ± 0.1856
ViT-B16 + AuxNet	0.8588 ± 0.0520	1.0534 ± 0.0686
ViT-B32 + AuxNet	0.8783 ± 0.0639	1.1386 ± 0.0759

Table S3. Results of the cross-validation (mean +/- stdev over the 5 folds) without AuxNet.

Model	Validation data	
	aMAE	aRMSE
ResNet101	0.8489 ± 0.0624	1.0388 ± 0.0696
Inception-v3	0.8749 ± 0.0538	1.0815 ± 0.0634
EfficientNet-b0	0.8326 ± 0.0867	1.0260 ± 0.1009
EfficientNet-b1	0.8854 ± 0.0562	1.0880 ± 0.0683
EfficientNet-b2	0.8485 ± 0.0854	1.0446 ± 0.1022
EfficientNet-b3	0.8868 ± 0.0568	1.0876 ± 0.0695
EfficientNet-b4	0.8309 ± 0.0574	1.0514 ± 0.0989
EfficientNet-b5	0.9165 ± 0.0676	1.3785 ± 0.5747
ViT-B16	0.8370 ± 0.1193	1.0468 ± 0.1037
ViT-B32	0.8860 ± 0.0524	1.0852 ± 0.0712

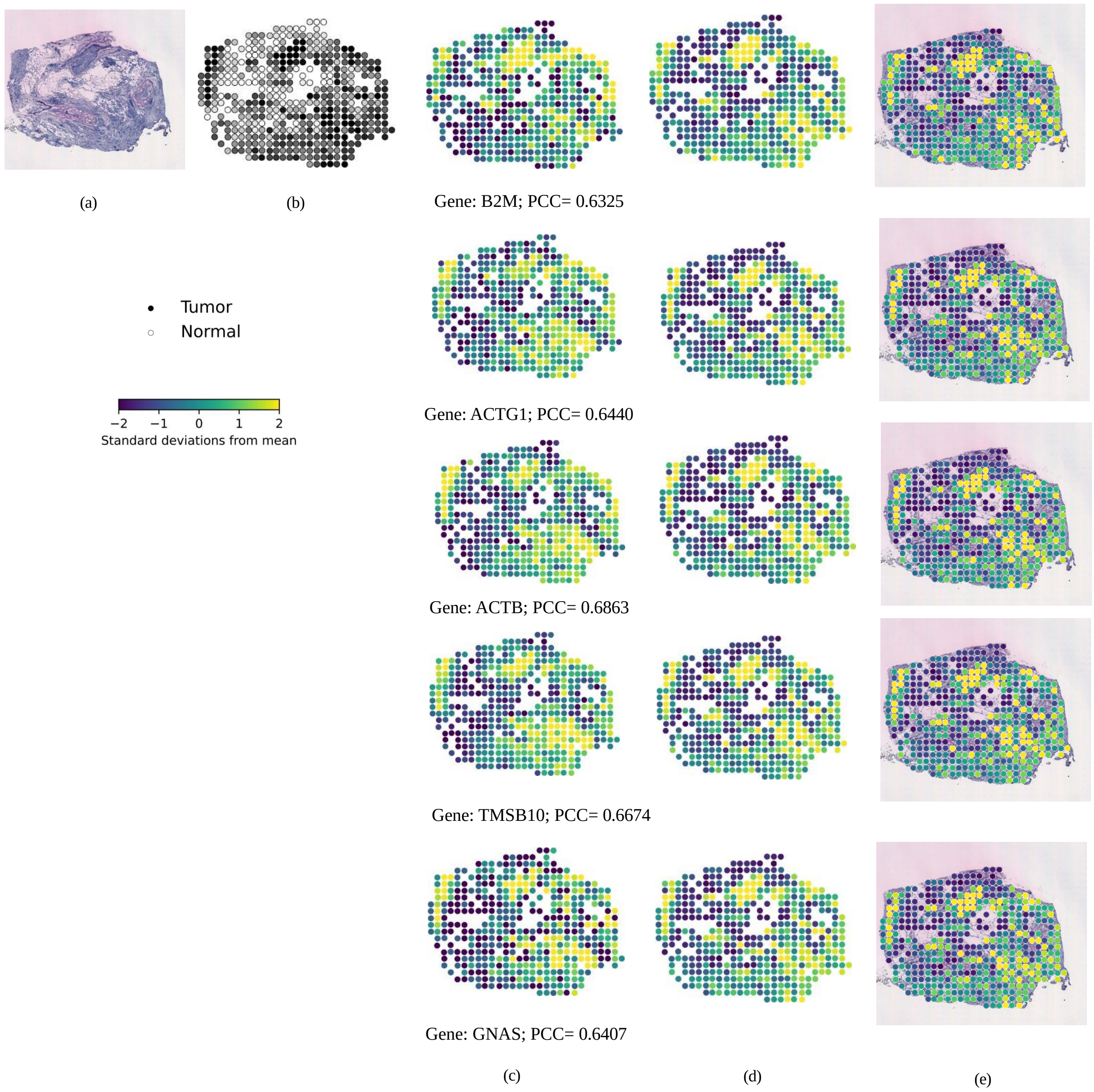

Figure S1. Visualisation of gene expression for patient BC23903 slide number C2. (a) Histopathology sample from the test data. (b) Binary label of tumour (black) and normal (white) areas, with gray parts indicating areas that are not purely tumor or normal. (c) Ground truth expression of corresponding genes. (d) Predicted expression of corresponding genes and their PCC values (e) Visualisation of predicted gene expression overlayed on the tissue slice.

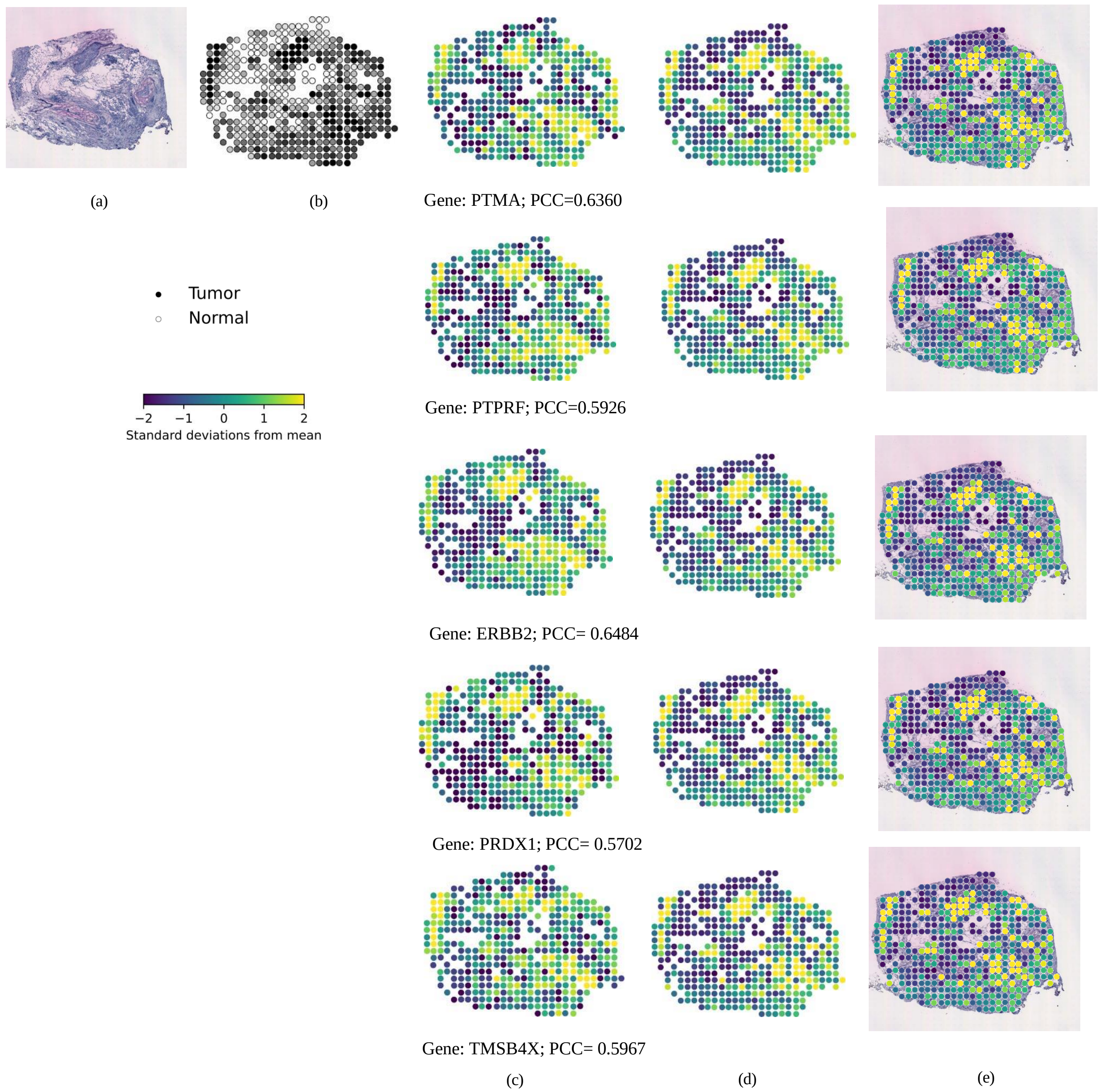

Figure S2. Visualisation of gene expression for patient BC23903 slide number C2. (a) Histopathology sample from the test data. (b) Binary label of tumour (black) and normal (white) areas, with gray parts indicating areas that are not purely tumor or normal. (c) Ground truth expression of corresponding genes. (d) Predicted expression of corresponding genes and their PCC values (e) Visualisation of predicted gene expression overlayed on the tissue slice.
